# Reconsidering the *Toxoplasma gondii* interactome: opportunities beyond crosslinking mass spectrometry

**DOI:** 10.1128/mbio.02683-25

**Published:** 2025-10-09

**Authors:** Nathkapach K. Rattanapitoon, Patpicha Arunsan, Nav La, Schawanya K. Rattanapitoon

**Affiliations:** 1 Parasitic Disease Unit, FMC Medical Center, Nakhon Ratchasima, Thailand; 2 Faculty of Medicine, Vongchavalitkul University 155164, Nakhon Ratchasima, Thailand; 3 Faculty of Pediatrics & Medicine, International University 231162, Phnom Penh, Cambodia; Tsinghua University, Beijing, China

**Keywords:** *Toxoplasma gondii*, interactive, crosslinking mass spectrometry, machine learning, dense granule proteins

## LETTER

Tomita and colleagues (1) have presented a technically impressive map of the *Toxoplasma gondii* interactome, integrating crosslinking mass spectrometry (XL-MS) with machine learning. This study provides one of the most systematic attempts to probe protein–protein interactions in *T. gondii* and deserves recognition for bridging structural proteomics with computational modeling.

While the work clearly advances the field, we believe its impact will be maximized if three broader issues are addressed.

The problem of proteomic “visibility”: as the authors acknowledge, their cytosolic data set favors abundant proteins such as ribosomal and proteasomal components. Yet, the parasite’s biology—and its persistence in chronic infection—is often driven by molecules expressed at low levels or in specific life-cycle stages. Previous proteomic maps, including hyperLOPIT (2) and ToxoNet (3), highlight proteins with restricted expression patterns that are not captured here. Combining XL-MS with fractionation strategies or bradyzoite cyst culture systems (4) may reveal interactomes linked directly to immune evasion, latency, and reactivation. Such an approach would shift XL-MS from descriptive mapping toward explaining clinically relevant phenotypes.

Machine learning, from validation to discovery: the introduction of LightGBM into XL-MS analysis is an important step. However, the risk of circularity remains when training on resources like STRING or hyperLOPIT, which already encode well-characterized interactions. This design tends to reinforce consensus complexes rather than expose the unexpected. We suggest that future models incorporate deliberately uncurated or orthogonal data sets—for instance, stage-specific transcriptomic perturbation or CRISPR fitness landscapes (5). By doing so, machine learning could transition from confirming known complexes to discovering novel, parasite-specific assemblies.

Dense granule proteins as a window into pathogenesis: perhaps the most intriguing finding is the appearance of dense granule protein (GRA) networks in “cytosolic” preparations. The authors attribute this to vesicular contamination, but an alternative view is that these interactions reflect transient trafficking intermediates. GRAs, such as GRA7 and GRA17, are central to vacuole remodeling and nutrient acquisition (6, 7). If XL-MS can capture these fleeting assemblies, it may provide the first structural entry point into how *T. gondii* modulates host cell permeability and establishes chronic infection. Applying this strategy to bradyzoite cyst wall proteins could yield insights directly relevant to therapeutic resistance.

In conclusion, Tomita et al. (1) deliver a technically rigorous interactome map that will be widely used by the community. Its true potential lies in extending the approach to less accessible proteomes, leveraging unbiased computational features, and focusing on parasite structures with direct clinical significance. Doing so may transform XL-MS from a methodological advance into a platform that shapes our understanding of host–parasite biology and drug development.

## Data Availability

This article does not report any new data.
